# “You Feel They Have a Heart and Are Not Afraid to Show It”: Exploring How Clients Experience the Therapeutic Relationship in Emotion-Focused Therapy

**DOI:** 10.3389/fpsyg.2019.01996

**Published:** 2019-09-04

**Authors:** Øystein Ottesen Nødtvedt, Per-Einar Binder, Signe Hjelen Stige, Elisabeth Schanche, Jan Reidar Stiegler, Aslak Hjeltnes

**Affiliations:** ^1^Department of Clinical Psychology, Faculty of Psychology, University of Bergen, Bergen, Norway; ^2^Norwegian Institute of Emotion-Focused Therapy, Bergen, Norway

**Keywords:** emotion-focused therapy, the therapeutic relationship, thematic analysis, depression, self-criticism

## Abstract

**Background:** The therapeutic relationship is regarded as an important source of change in emotion-focused therapy (EFT) (Greenberg, 2014; Watson, 2018), but few qualitative studies have specifically investigated how clients experience the role of the relationship in EFT.

**Aim:** The purpose of the present study was to explore what clients experienced as helpful or hindering aspects of the therapeutic relationship when undergoing EFT for depression, anxiety, and severe self-criticism.

**Methods:** We interviewed 18 clients after a clinical trial of time-limited EFT, to explore their experiences of the relationship with their therapist during treatment. The interviews were analyzed using hermeneutic-phenomenological thematic analysis (Braun and Clarke, 2006; Binder et al., 2012).

**Results:** We identified four main themes: (1) Forming a trusting relationship or finding it hard to connect, (2) Collaborating and struggling to find new ways to relate to painful feelings, (3) Alliance ruptures and needs for repair when working with distressing emotions, and (4) The significance of new relational experiences.

**Conclusion:** Clients described therapists’ genuineness and the establishment of trust in the relationship as important preconditions to open up to vulnerability and painful feelings, and engaging fully in specific EFT interventions. The findings also indicate that the therapists need to be mindful of different client preferences and monitor potential alliance ruptures when working to change distressing emotions in therapy.

## Introduction

What is it like to open up and reveal your most vulnerable and painful feelings to another person when you are struggling with depression, anxiety, and self-criticism? How do clients experience the role of the relationship with their therapist when undergoing a time-limited emotion-focused therapy (EFT)? The therapeutic relationship is considered by many leading clinicians and theorists to be the main vehicle for client change (Rogers, 1951/2012; Sullivan et al., 1955/2003; Mitchell, 1993; Greenberg, 2007). A growing body of scientific research has also found that the quality of therapeutic relationship is an important predictor of client outcome (Horvath, 2000; Lambert and Barley, 2001; Norcross and Lambert, 2018), with Lambert and Barley (2001) arguing that that the nature and quality of the therapeutic relationship is *the* most significant curative factor in psychotherapy. The evidence base for these claims is mainly based on quantitative studies, with some notable exceptions. Existing qualitative studies have consistently found that clients appreciate an understanding, warm, caring and engaged therapist (Orlinsky and Howard, 1967; Toukmanian and Rennie, 1992; Levitt et al., 2006; Timulak, 2007; Israel et al., 2008; Mortl and Von Wietersheim, 2008; Watson, 2018). Previous research on the clients’ perspectives particularly point to the importance of the caring and supportive relationship (Labott et al., 1992; Timulak and Elliott, 2003; Levitt et al., 2016; Timulak and Keogh, 2017; Timulak et al., 2017). In a meta-analysis of 109 qualitative studies of clients’ experiences of psychotherapy, Levitt et al. (2016) found that authentic caring from the therapist allowed clients to feel validated and engage in vulnerable discussion, but that overinvolvment could also hinder client’ sense of agency. Timulak and Keogh (2017) highlight that clients emphasize the warmth, authenticity, honesty and dedication of the therapist during the formation of the therapeutic relationship. In this article, our main focus is to investigate how clients experience the role of the therapeutic relationship within the context of EFT. We investigate what clients experience as helpful and hindering aspects of the therapeutic relationship when undergoing EFT for depression, anxiety, and severe self-criticism.

Emotion-focused therapy is a humanistic psychotherapy that emphasizes helping people to access and change their emotional experience (Greenberg et al., 1993; Greenberg and Watson, 2006; Greenberg, 2015, 2017). The aim in EFT is to access and transform maladaptive emotions (i.e., overgeneralized fear and shame) that are hypothesized to be the source of depressive symptoms, and mobilize adaptive emotions (i.e., assertive anger and sadness over losses) that promote growth and therapeutic change (Greenberg and Watson, 2006; Greenberg, 2017). EFT integrates interventions from person-centered therapy and gestalt therapy, combining an affectively attuned empathic relationship and chair work interventions to access and transform specific emotional processes that contribute to anxiety and depressive symptoms. The task of developing an empathic relationship characterized by presence and affective attunement is central to the hypothetical mechanisms of change in EFT, and considered to have ultimate priority over and beyond specific psychotherapeutic interventions (Greenberg, 2014). Greenberg and Watson (2006) argue that the therapeutic relationship in EFT serves two primary functions by providing interpersonal regulation of affect, and creating a safe atmosphere for inner exploration and transformational work with emotions.

Although a growing body of quantitative research have investigated the impact of the therapeutic alliance in psychotherapy in general and EFT in particular (Norcross and Lambert, 2018), there is in comparison fewer qualitative studies with an emphasis on exploring clients’ own first-person experiences of the therapeutic relationship in EFT. Some qualitative studies have explored the clients’ experience of EFT have found that clients describe the therapeutic relationship as important in their process of change. Timulak et al. (2017) recently conducted an exploratory mixed methods study of EFT for 14 clients with GAD, reporting that the clients described the helpfulness of a soothing and validating relationship with the therapist, being listened to and understood by the therapist, and being able to share experiences with the therapist. Studies that have investigated significant events using used brief structured recall have also found that clients emphasize relational aspects of therapy (Labott et al., 1992; Timulak and Elliott, 2003). Hermeneutic single-case efficacy design have also found that clients described acceptance or unconditional positive regard in their relationship with their therapist as important in process-experiential or EFT (Stephen et al., 2011; MacLeod and Elliott, 2014). Previous qualitative research on EFT have found that clients emphasize the role of the therapeutic relationship. The studies of clients’ perspectives, however, have often been conducted with a broader focus on EFT. To the best of our knowledge, the existing studies have not had a specific focus of how clients experience the therapeutic relationship itself in the context of EFT. Qualitative studies may be important to form new empirical knowledge that can only be acquired by exploring clients’ idiosyncratic experiential worlds (Elliott and James, 1989; Binder et al., 2012). Exploring how clients experience the process of therapy can for instance serve to investigate the relevance of specific interventions, provide sources for new research questions, and in general discover phenomena not easily investigated by quantitative methods (McAleavey and Castonguay, 2015).

The aim of this study was to explore how clients experience the role of the therapeutic relationship in EFT. The participants in this study were drawn from a larger clinical trial of time-limited EFT as a return to work intervention for individuals struggling with major depression, anxiety, and self-criticism (Stiegler et al., 2018b). We have previously examined how clients experienced the process of undergoing two-chair dialogues to work with their emotions (Stiegler et al., 2018a) and their experience of pre-determined time-limits as context for therapeutic work (Submitted). In the present article, we investigated the role of the therapeutic relationship during the different phases of the therapeutic process, by exploring how clients’ in retrospect conceptualize and experience the therapeutic relationship over the course of therapy. We explore the following main research question: what do clients with depression, anxiety, and self-criticism experience as helpful or hindering aspects of the therapeutic relationship when undergoing EFT?

## Materials and Methods

### Participants

The participants, all native Norwegians, were recruited from a public RTW program designed to treat mental health conditions that resulted in sick leave (Stiegler et al., 2018b). Eligible patients suffered from mild to moderate severity of anxiety and/or depression and had to meet criteria for mental health difficulties as assessed by an intake interview. As this particular trial had a specific focus on the reduction of self-criticism, participants had to display moderate to high self-criticism to be included as measured using Self-Criticizing/Attacking and Self-Reassuring Scale (Gilbert et al., 2004). Of 24 participants, twenty-one completed the treatment. Out of 21 completers, 18 participants (13 women, aged 20–63, mean age 38.2 years) accepted to be interviewed post-treatment (Stiegler et al., 2018a).

### Treatment

The study was based on a multiple baseline design, where the treatment involved two distinctive phases. The first phase emphasized empathic attunement to affect based on Rogerian principles (Rogers, 1951/2012). The therapists subsequently implemented chair dialogue interventions in the second phase. Participants were given between 5 and 9 sessions of baseline treatment in the first phase, followed by five sessions with the two-chair dialogue in the second phase. Six therapists delivered the treatment. All therapists were clinical psychologists with 5–13 years of clinical experience (mean 9.2 years). Four therapists were female, and two were male. The therapists had a minimum of 300 h of EFT training over at least 3 years, and a minimum 20 h of supervision on their recorded practice.

### Methodological Approach

We conducted semi-structured qualitative interviews, and used a hermeneutic-phenomenological approach to thematic analysis. This approach seeks to interpret (hermeneutical) and explore lived experience (phenomenological) by systematically investigate people’s descriptions of their subjective experiences and life worlds. This hermeneutic-phenomenological approach recognizes that the researchers’ own background, preconceptions, and assumptions will inevitably influence the interaction with the informants and the interpretation of data in the research process (Binder et al., 2012), highlighting the need for researcher reflexivity (Finlay and Gough, 2003).

### Data Collection Method

The interviews were conducted between August and September, 2015. Due to practical availability, we conducted the interviews with the participants approximately 3 months after treatment. The strategy of interviewing all available participants who completed the trial was set in advance, as we estimated that at least 10 informants would be necessary to ensure a sufficiently rich data material. The interviews were based on a semi-structured interview guide, and explored participants’ motivations for and experiences in therapy, experiences about the therapeutic relationship, and experiences with chair dialogue interventions. Examples of questions from the interview guide include: can you tell us about how you experienced the first meeting with your therapist? How did you experience the contact with the therapist? Can you remember a situation of therapy that was difficult or challenging for you? When you look back, what is the most important change that you experienced? The interviews were audio recorded and transcribed verbatim by eight graduate students supervised by the last author.

### Data Analysis

We analyzed the transcribed material using a hermeneutic-phenomenological approach to thematic analysis (Braun and Clarke, 2006; Binder et al., 2012), and proceeded through several phases. First, the research team familiarized themselves with the data material. Second, the first author examined the material in detail and coded relevant units of meaning based on the research question using Nvivo 11 (QSR International, 2015) software. We examined the parts of the interview guide focusing on the clients’ experiences of the therapeutic relationship. Third, the first author constructed tentative themes by sorting identified units of meaning in categories in collaboration with the second and last author. Fourth, the tentative themes were reviewed, modified and redefined to create a coherent narrative of the main findings. The first, second and last authors had regular meetings to discuss different interpretation of findings and identify the main themes. For example, we discussed how to understand clients’ different experiences of therapists’ empathy, and alliances ruptures during treatment. Fifth, the findings were reviewed and critically audited by the third, fourth and fifth authors. The research team met to discuss different interpretations of the data, for example regarding how to understand the challenges the clients described. Sixth, the newly modified themes were reviewed by the first, second, and last author. Seventh, the first and last author conducted a cross-analysis of the themes to examine the frequencies of these themes in the data set. Finally, the main findings and interpretations were consensually discussed with the research team for final agreement.

### Researchers

The first author is a clinical psychologist with a special interest in experiential and dynamic psychotherapy. The second author is a professor of clinical psychology with 23 years of clinical experience, 3 years of training in EFT and a special interest in qualitative and mixed-methods research on psychotherapy. The third author is an associate professor in clinical psychology with 11 years of clinical experience and a special interest in the first person perspective of therapy, change processes, and recovery. The fourth author is a clinical psychologist and researcher with a special interest in emotional processes in psychotherapy and EFT training. The fifth author is associate professor in clinical psychology with 17 years of clinical experience, and a special interest in psychotherapy process, mindfulness, and deliberate practice. The last author is an associate professor in clinical psychology with 3 years of training in EFT and a special interest in empathy, process research, and psychotherapy integration. All authors share and interest in humanistic perspectives, qualitative methods, and psychotherapy process research. To promote a balanced investigation as possible, the first, third. and fifth authors who do not have a professional background with EFT represented important outsider perspectives on the data material during the research process.

### Ethics Statement

This study was approved by the Norwegian Regional Committees for Medical and Health Research Ethics (REK West, Project code: 2014/1618). The participants were given written information about the study, and voluntarily informed consent was obtained from all participants before treatment. The data was handled and stored safely in accordance with protocols from the Norwegian Regional Committees for Medical and Health Research Ethics.

## Results

We identified four main themes through the analytic process: (1) Forming a trusting relationship or finding it hard to connect; (2) Collaborating and struggling to find new ways to relate to painful feelings; (3) Alliance ruptures and needs for repair when working with distressing emotions; and (4) The significance of new relational experiences. The themes have a chronological structure, where the two main first themes describe both the helpful and challenging experiences the participants described within the initial phase (theme 1) or working phase (theme 2) of the therapeutic process, and the final main themes illustrate how the participants subsequently described these experiences as leading to the challenging experiences (theme 3) and the positive experiences (theme 4) in the therapeutic relationship as therapy progressed. We will describe these themes in more detail below. The frequencies of these findings are presented in Table 1.

**TABLE 1:** Frequencies of the thematic categories.

	***N***	**%**
(1) Forming a trusting relationship or finding it hard to connect	17	94.44
(2) Collaborating and struggling to find new ways to relate to painful feelings	13	72.22
(3) Alliance ruptures and needs for repair when working with distressing emotions	12	66.66
(4) The significance of new relational experiences	8	44.4

### Forming a Trusting Relationship or Finding It Hard to Connect

This first theme describes clients’ experiences of their initial contact with the therapist. Many participants reported that being met by an interested, caring and present therapist created a safe space where they could open up and reveal their vulnerability and personal struggles, which helped them to form a trusting emotional connection in the beginning of the therapy. One participant described how she experienced the first encounter with the therapist:

She was nice, smiling, and comforting. I had her attention and got to explain. She asked sensible questions which enabled me to talk about the things I needed to talk about. The best way to put it is that she was there for me.

This quality of “being there for me” was highlighted by several participants. They emphasized the importance of being met by a genuine person who related to them in an honest and authentic way, without any sense of judgment. One participant described how she experienced these qualities in her therapist:

Certain people have that kind of skill independent of profession. Some people are genuine, and you notice they are not judging and have tolerance for different viewpoints. They have presence and a warm gaze. You feel they have a heart and are not afraid to show that they have it. And that they care.

The therapist’s good intentions and genuine interest was also inferred by therapist behavior, like remembering information between sessions:

I noticed that he quickly learned the names of the important people in my life. Coming to a session where he had complete overview of the whole gallery in his mind made it possible to get to work faster. It confirmed that he actually listened to me. That is important to me – to be remembered.

For some participants the therapist’s emotional reaction to the things they shared was also an important aspect in establishing a sense of trust in the relationship:

I was listened to. I felt that when I cried, she almost sat there and cried together with me. I felt there was a strong connection, which made it easy for me to open up. Even though I was prepared to talk about my problems, still, it became even easier. Safety to open up, and there was something about how the therapist acted. I feel I have been receiving a lot from her. She was there for me. It has been very close and nice, and very supportive. Very safe.

Although most clients described feeling seen and validated by their therapist, some reported that they also had interpersonal needs and personal expectations for the therapy that were not fully recognized or sufficiently addressed by the therapist. For instance, two participants experienced the empathic responses of the therapist as excessive, and described that they had hoped for a more challenging therapist:

I thought the first session was a bit awkward. I felt I got too much sympathy for my problems. I had perhaps hoped that I got someone who could challenge me more. After the first session I thought ‘Oh my God, this is not helpful at all.’ I had not intended to have someone sitting next to me and patting me on the back. That is not what I need.

Some participants also felt a bit impatient in the beginning of the therapy, and expected more direct work with emotions early on. In retrospect, they realized that the foundation built in the beginning was crucial for the continuation of therapy: “I see now that it was necessary to spend such a long time to build trust. If not, then I don’t think I would have dared to let go and participate in the manner that I did.”

### Collaborating and Struggling to Find New Ways to Relate to Painful Feelings

The second main theme describes participants’ experiences of working to change difficult emotions in collaboration with the therapist and highlights the importance of having established a safe relationship in order for participants to dare opening up to painful feelings. As those feelings were expressed and brought out into the open, participants described how the therapists’ acceptance of those feelings provided them with a new way to relate to their feelings as less dangerous and scary:

I came in contact with an inner space of feelings within me that had never been opened before. It was very scary to let it out. I said to him that it felt like I fell through the earth and into a huge, dark room. I don’t think that I would have been able to open up in that way, even though I was prepared for it beforehand, had it not been for the safe relationship with the therapist.

Another participant experienced that how the therapist reacted to the expression of vulnerable emotions helped establish a sense of trust where it was safe to show these feelings, making the participant more fully engaged in and committed to experiencing and expressing their own feelings:

You feel that you can participate a bit more than you otherwise would because you have that trust. If I am really sad, she does not look at her watch, or try to say ‘you have to pull yourself together.’ I had quite a trusting relationship with her.

Furthermore, the safe connection with the therapist provided the participants with a sense of not being alone with their pain. One participant mused: “I think it had something to do with not being alone with your emotions – that somebody is there and sees it.” Several participants had initially regarded their own emotional reactions as embarrassing, irrational, or had a sense of not being entitled to feel them. One participant stated: “I had an idea of what the problem was, but you got to allow yourself to feel it. That it is okay and important what I feel. This therapy actually allowed me to feel.” For this participant, the relationship with her therapist helped her to be able to allow herself to approach and accept feelings she had not felt before.

Participants also found the manner in which the therapist contextualized their emotional reactions as very important, allowing them to realize that it was understandable that they felt in a particular way in response to difficult circumstances, rather than due to some personal deficit, fault, or abnormality. Having one’s emotions contextualized as natural and understandable allowed participants to view themselves differently – as being part of a common humanity who suffers: “My feelings were normalized – it is not just me who thinks and feel in these ways. When you feel there is nothing wrong with you, it becomes less scary.”

In addition to being helped to view themselves as normal, participants highlighted the significance of experiencing the therapist’s explicit validation of their emotions and the therapists’ compassionate recognition and interpretation of non-verbal expressions:

He somehow picked up on my facial expressions and told me like ‘Now I see that you are sad’ such that it helped me put the feeling into words. Then I would confirm whether it was correct or not, and many times it was. It was just that I was not able to express it first by myself. He validated what I said by saying for instance ‘I perfectly understand that you are angry right now, just get it out, say what you really think.’ He also noticed my body language – something I cannot observe myself – including shifts in posture. This helped in thinking about how I felt.

However, some participants expressed that they withheld some of their self-critical thoughts from the therapist when describing their negative self-statements, or felt a need to excuse themselves for their thoughts as they found those too embarrassing. At times, severely harsh self-critical thoughts could be too difficult to disclose to the therapist:

I felt that I held some back from my therapist. You know, my thoughts are worse than what I am able to express. I can’t get a complete accordance between what I say to myself in my thoughts and what I say aloud. On their way out, I realize how bad they are and I hold back. Almost sort of embarrassing. Also to admit how bad it is.

This example shows that explicit focus and dedicated work to establish a trusting therapist-client relationship is not always sufficient to promote disclosure and exploration of emotional difficult themes.

### Alliance Ruptures and Needs for Repair When Working With Distressing Emotions

The clients experienced that the challenges of approaching distressing emotions could itself create difficulties in the therapeutic relationship. During the course of therapy, participants also experienced challenges in the therapeutic relationship, including misunderstandings, disagreements, and/or concerns that were not sufficiently addressed in therapy. This included disagreements on how to understand the participants’ problems and how to address them such that they became obstacles in treatment. In some cases, the participants and their therapists worked out these difficulties. However, in other cases, difficulties with expressing such concerns to the therapist prevented resolution and common understanding.

Some participants experienced that their therapist’s conceptualization of their problem differed from their own understanding, thus feeling misunderstood:

I think she perceived me to be cruel to myself, but I am really not. So we misunderstood each other a bit. … I thought it was a bit unfair as I was not that hard on myself. But we worked it out – we found out how I was.

In another cases of misunderstanding, the therapist’s emphasis and suggestions were in discord with the client’s goals, leading the client to feel that the therapist’s suggestions did not seem viable:

I talked to my therapist about it, because I felt for each thing she helped me figure out I got an even bigger question which I wondered about. We talked a little about that I had to strive less. However, at the same time it felt unnatural for me. I thought: ‘I have to get back to work, I can’t just sit on the sofa and notice that the anxiety is tearing me apart and I get more hopeless about the future.’ I can’t sit there and just think ‘but I am going to the therapy in 5 days.’ I felt I had to do something. I couldn’t just be passive and watch. It is difficult to not work too. What am I supposed to do? Just sit down and be afraid?

Another type of challenge that some participants experienced in the therapeutic relationship revolved around the notion of there not being enough space for what they needed to talk about in the sessions:

I remember I had many questions and difficulties regarding a choice I had to make, and I could not tolerate my anxiety. I needed to talk about ways to manage it. … And to talk about what I could do the rest of the week. There were multiple times I very clearly understood that we were now supposed to switch topics, as if the therapy had been arranged before I arrived. What we worked on was helpful, but it was not necessarily what I needed the most at that time.

Furthermore, six participants expressed that they expected a more structured treatment from the start, including homework and assignments between the sessions and concrete advice on how to manage anxiety, negative thoughts, and difficult situations between sessions:

I needed a technique or a recipe or a safety net I could cling to when the anxiety was at its worst – whether it was a breathing exercise or to walk 10 steps back and forth. It is so scary to be paralyzed and terrified by anxiety, and then one is supposed to dive into it without. I can dive into it if someone promises to catch me so that I get back up – if I can hold on to that technique and know what I am doing when I am shaking. I just become so desperate just sitting down and notice that I am in such pain.

Some participants also found it challenging to address aspects of the therapy that did not work well with their therapist. In one case, a participant was unable to express that the way the therapy unfolded was not in line with her expectations. What was most important for her to talk about was therefore not addressed.

I was perhaps not clear enough or good enough to address it and say that it was not completely in line with my expectations. So there I could have been clearer. … If you feel it is not as helpful, it is somewhat wasted. When it requires such energy, it is a pity if in the end you don’t feel that one has shared what one thought was most important beforehand.

### The Significance of New Relational Experiences

Several participants described that being met in a particular and novel way when feeling vulnerable was a very profound and meaningful experience:

He was with me, when you are kind of on the other side of the bridge. He gave much support as to the reality of [my difficulties] and how understandable they were. When those closest to you disappear. how it impacts on the way you understand yourself and those things. He went into it and started to normalize my reactions. They had to go somewhere, he said. It has to come out somewhere. Now, it was a kind of bridge building.

Such experiences were often felt as new and corrective compared to how they had been met by others in the past.

The support he gave when we talked about my sexual orientation that session affected me deeply. An incredibly good feeling, you know, a person I really experienced actually managed to understand my despair – how it was for me. That sticks out. It was such a contrast to the attitudes I remembered. And then you get this kind of recognition on these matters. I had not even thought beforehand that this topic would come up.

This also included empowerment to express new feelings, through the therapist’s encouragement of expressing assertive anger:

One time when the session was over and I was on my way out, we talked about something and then I say ‘Damn!’ And then she said ‘Say it again!’ (laughter). That was one of the times I really showed some anger (laughter). Yes, get it out. Get angry, say it one more time. That was an important episode.

For some participants, experiences of being validated as having worth and value lead to empowerment and real behavioral changes in life:

He helped me to confirm to myself that I am allowed to be myself, and that I am not supposed to be self-critical and put myself down, but feel good inside. To get confirmation that I am worthy of an hour break, I am worthy of saying no and to take care of myself. Getting that confirmation in the room. Standing up for myself – that was the turning point – putting it into words together with that support from him.

Many participants thus carried with them specific, vivid, and memorable moments in their relationship with the therapist that they experienced as meaningful and empowering during the therapy.

## Discussion

In this study, we explored what clients experienced as helpful and hindering aspects of the therapeutic relationship when working with emotions in the context of EFT for depression, anxiety, and self-criticism. The first theme, “Forming a trusting relationship or finding it hard to connect” can be understood as describing how the therapist in the initial phase of treatment sought to create optimal condition for inner exploration, by relating to them in a genuine way with warmth, authenticity, non-judgment and interest and gradually building a safe atmosphere for exploring frightening and painful emotions. Previous studies indicate that clients often attribute a good outcome to positive characteristics of the therapist (Sloane et al., 1975; Orlinsky et al., 1994; Lambert and Barley, 2001). Bordin (1979) highlighted that “some basic level of trust surely marks all varieties of therapeutic relationships, but when attention is directed toward the more protected recesses of inner experience, deeper bonds of trust and attachment are required and developed” (p. 254). Levitt et al. (2016) found that authentic caring from the therapist was important in allowing clients to feel validated and engage in vulnerable discussion, but that overinvolvment from the therapist could also hinder client’ sense of agency. Within the framework of EFT, such a bond is thought to create a therapeutic atmosphere where clients feel safe enough to explore their inner world with the therapist making new learning and emotional change possible (Greenberg, 2007). Participants’ descriptions of being recognized as a person and having their pain seen by another person, as opposed to only a professional, helped them to feel safe to open up and share their vulnerability. The experience of meeting a real and genuine person as equals who is present to bear witness to one’s inner pain may resonate with Gelso (2002) concept of the “real relationship.” The findings also indicate that some participants were expecting a more solution-oriented focus on their problems. This may reflect a conflict of core values where the emotionally-focused therapist stress that change has to come from within, while the participants may not have shared these assumptions or sufficiently understood the practical rationale for working in this way with emotional problems.

The second theme, “Collaborating and struggling to find new ways to relate to distressing feelings,” describes how the safety established during the formation of the therapeutic relationship enabled participants to dare approaching intense and painful emotions. The findings indicate how the manner in which the therapist responded to the expression of feelings was important for how the participants subsequently related to their own feelings, and provided a new self-understanding. Several participants explicitly stated that how they felt in the relationship with the therapist was paramount in their ability and willingness to continue explore and feel disavowed, scary and painful feelings – including a space within of feelings that had “never been opened before.” The participants often mentioned normalization as an important therapist behavior. Normalization is a general term that has been understood as referring to ways of depathologizing clients and change the manner in which they view themselves and their feelings from abnormal to normal (Corcoran, 2002). In the context of EFT, the communication of empathic understanding and confirmation from the therapist is seen as important for the clients’ awareness and acceptance of themselves and their experiences (Elliott et al., 2004). In the context of how the participants in our study used this term, it may refer to the experience of being empathically validated and supported in gaining an accepting sense of self where one does not feel shame due to one’s feelings. Normalization may thus often mean contextualization of feelings as natural and valid reactions to hardship and stressful circumstances, instead of feelings due to personal insufficiency. By understanding this, participants could become increasingly aware that they are part of a common humanity, as opposed to realigning themselves on the socially constructed dichotomy of normal-abnormal. The therapist modeled and conveyed acceptance for the participants as human beings with understandable emotional reactions.

The findings from the second main theme resonate with the conceptualization of the therapeutic relationship in EFT as an affect-regulating bond (Greenberg, 2014). Several participants highlighted the experience of being witnessed and having one’s emotions explicitly validated by the therapist, helped them to both verbalize and become more aware of their feelings. Being aware of one’s emotions, including the ability to recognize and label one’s emotions, is considered to be an important component in emotion regulation (Moyal et al., 2013). Although many participants felt safe enough in the relationship to increasingly express themselves in an authentic manner, some participants still held back their thoughts from their therapist due to shame. For instance, particularly harsh self-critical thoughts were deemed as too embarrassing to share with the therapist by one participant during inner critic chair work. Our findings suggest that clients can withhold important negative experiences from the therapist during chair work in EFT. In general, holding back relevant experiences from the therapist is not an uncommon phenomenon in psychotherapy (Rennie, 1994), which may have both beneficial and potential harmful consequences. Not disclosing negative information could represent a way for clients to collaborate with their therapist, but may also make it more difficult for therapists to detect alliance ruptures and negative treatment effects in therapy.

The third theme, “Alliance ruptures and needs for repair when working with distressing emotions” describe how alliance ruptures may also occur in EFT as part of working to change distressing emotions in therapy. Participants’ experienced challenges in the therapeutic relationship related to misalignment between the therapist’s and participant’s understanding of the problem, and how to use the time in the sessions. What felt most important and pressing for participants in a given session were not always picked up by the therapist. In addition, explicit disagreements about the nature of participants’ problems occurred. This may not solely represent an EFT issue but might have to do with lack of certain skills on the therapist side when the patient presented issues that called for more active or directive interventions. Such instances may also reflect challenges in the relationship that concerns experiential horizons that do not fully meet and fuse, and possibly conflicts about core values (Stolorow et al., 2008; Binder et al., 2012). In their qualitative meta-analysis, Levitt et al. (2016) found that clients feeling unheard, misunderstood or unappreciated can potentially challenge the therapeutic alliance and require discussion of differences. Alliance ruptures involve a “tension or breakdown in the collaborative relationship between patient and therapist” (Safran et al., 2011, p. 80). Alliance ruptures can range from minor and vague to dramatic and pervasive, and can occur in all phases of therapy (Safran et al., 2011). In EFT where a main task is to encourage exploration of inner experience (Elliott et al., 2004), clients can be reluctant to do this, or not understand the rationale properly. This may reflect the therapeutic format in EFT, or represent an issue of particular therapists and their specific way of working. Some statements by the participants in this study indicate that there occurred both minor and major ruptures that were either not addressed by the therapist or disclosed by the client. For instance, some participants wanted sometimes to focus on other topics than self-criticism, which was the main focus of the larger clinical study. This may illustrate that some clients wished for more flexibility in the therapeutic focus so that their needs in therapy could be met. This is consistent with a recent study by Silberschatz (2017) finding that the degree to which the therapists were responsive to the clients’ own formulation of their problems and conflicts related strongly to outcome. However, the study design of the trial the present participants underwent predefined that two-chair interventions targeting self-critical work were going to be implemented after a predetermined number of sessions. The therapist had to withhold the self-critical chair interventions in the first phase of treatment, and then to use these interventions actively in the second phase of treatment. This may have limited the therapists’ flexibility to choose what problem they would respond to (client marker), how they would respond to this problem (task) and when they could use specific interventions to target particular clients problems (phase one or phase two of the multiple baseline design) (Stiegler et al., 2018b). The present study does, however, indicate that alliance ruptures and repair processes may be important to understand how the therapeutic relationship may hinder or promote change in EFT. It is important to note that while many of the participants disclosed alliance ruptures, and that many of these were not detected and by the therapist, all participants described the therapeutic relationship as important in their process of change.

The final theme, “The significance of new relational experiences” describes experiences in the therapeutic relationship that participants described as particularly significant and healing during their therapy. A common theme in the findings was that participants experienced being met and related to in a novel and unexpected manner when showing their deepest vulnerability to the therapist, providing participants with a new way of relating to themselves. These experiences and may be considered as examples of “corrective emotional experiences” where participants had were met with acceptance and confirmation in contrast to negative experiences with other people in the past (Alexander and French, 1946; Castonguay and Hill, 2012). From an EFT perspective, a CE is an experience “in which a person has a new emotional response to an old situation,” enabling the expansion of the client’s emotional response repertoire (Greenberg and Elliott, 2012, p. 90). Our findings demonstrate examples of such corrective experiences, where participants got access to new-found adaptive anger after encouragement from the therapist which had meaningful positive real-life consequences. Corrective experiences are thought to be facilitated by a safe environment where the therapist modulates the intensity of buried feelings as they emerge (Greenberg and Elliott, 2012). The findings also describe specific experiences that particularly stood out for some participants where they were met with genuine acceptance for one’s whole being when they revealed themselves at their most vulnerable. The participants experienced that nothing bad happened when showing their inner feelings and this was described as very powerful.

The findings in this study, seeking to understand client’s experiences of helpful and hindering aspects of the therapeutic relationship in EFT, may be relevant for the more general debate in the psychotherapy research literature as to whether common factors are both necessary *and* sufficient, or if specific factors are also needed for optimal client outcomes (Wampold and Imel, 2015). Greenberg (2014) have argued that the therapeutic relationship serves a dual purpose in EFT: both as an end in itself and as a means to an end. Silberschatz (2007) proposed that although Rogers (1957) thought the relationship with its Rogerian qualities itself as sufficient for successful treatment, there are also clients “who require more technical approaches (e.g., interpretations, homework, relaxation techniques, mindfulness training, etc.)” (Silberschatz, 2007, p. 266). Participants’ experiences of the quality and nature of the therapeutic relationship in the present investigation may indicate that the relationship constitutes the context in which specific interventions are put into practice. The participants in this study highlighted the significance of establishing a genuine and affectionate relationship with their therapist. Many of the participants experienced that the real relationship itself was important in changing their sense of self and emotional pain. The emphasis given to the experiences of being genuinely met with empathy, understanding, and acceptance from a real person seems to reflect change due to Rogerian conditions. Some participants also seemed to have had corrective experiences when sharing deep vulnerability in the therapeutic relationship. However, the findings also suggest that the therapeutic relationship served as a context for change, where the safety that the relationship enabled participants to dare open up to disavowed painful feelings, letting go of control and voluntarily participate fully in the specific chair interventions. At the same time, the relationship could also potentially be experienced as having a potentially negative impact in the form of unaddressed alliances ruptures.

### Reflexivity

Reflexivity involves examining how the subjectivity and perspectives of the researchers may inform different stages of the research process (Finlay and Gough, 2003). In this study, it was important for us to consider how the professional background of several members on the research team might inform the research process. Four members of the research team have professional training in EFT, indicating the potential of researcher allegiance. The interview guide focused on experiential and emotional aspects of the participant’s experiences in therapy. Although the background of these researchers may have enabled a more precise investigation of the research question, it may have come at the expense of a more open query (Binder et al., 2012). To address this challenge, the research team included three researchers with no formal training in EFT. Also, the first author, leading the analysis, has no formal training in EFT. An important objective for the research team in the study has been to describe both the potentially helpful (positive) and challenging (negative) aspects of the relationship in EFT, where we sought to conduct an as open and balanced investigation as possible. We do recognize, however, a shared assumption in the research team that the therapeutic relationship is important in therapy, which may have impacted on the focus in this paper and the interpretation of these findings.

### Limitations

The present study has several methodological limitations. First, the qualitative research design does not allow inferences about causality. Second, the study investigated experiences a sample of 18 participants, the majority of them women, limiting the generalizability of the current findings. Third, due to termination date for the clinical trial (at summer vacation), the participants were interviewed approximately 3 months after treatment, which may have influenced how participants recalled events from therapy. A potential limitation in qualitative research is the potential for attributional errors when clients attribute positive changes to the therapy when they actually are the result of events outside the therapy, including life events, personal efforts, or biological changes (Elliott, 2002, 2010). We recognize that a case study approach would do more justice to the nuances and complex lived experience of each of the individual clients. Qualitative methods such as Interpersonal Process Recall (IPR) or hermeneutic single-case study designs (HSCSDs) might have be better suited to capture the complex moment-by-moment processes in the therapeutic relationships (Elliott, 2010). Fourth, it is possible that participants found it hard to be fully open about negative experiences during therapy in the interview context. Finally, although the trial included a naturalistic sample, the multiple baseline design prescribing a predefined structure with a different treatment course from ordinary structure of EFT, which may have given the therapist less flexibility in choosing how they would respond to clients’ problems. This may represent an important limitation in terms of ecological validity.

### Implications for Research and Clinical Practice

The present article has examined clients’ subjective experiences of the therapeutic relationship in EFT. To examine the transferability of the findings to other contexts, more in-depth qualitative studies of participants’ experiences in different contexts are needed. More in-depth qualitative studies of immediate client experiences of the therapeutic relationship in EFT, for example using IPR or hermeneutic single-case study designs (Elliott, 2010), may thus be important to complement this study and describe the complex moment-by-moment processes that contribute to the development of the therapeutic relationship. Future research should investigate how therapists can recognize and negotiate possible conflicts about values and preferences, and be more flexible within a therapeutic model. More research is also needed to investigate how alliance ruptures are experienced and negotiated by clients and therapists when working to promote transformative or dynamic emotional change, and what therapeutic strategies would be helpful to address these challenges.

The findings in this study may also have several implications for clinical practice. First, the findings suggest that it is important to spend sufficient effort and time to develop a relational safety and trust before implementing more emotionally demanding interventions. It may also be important that the clients do not feel pressured to undergo such interventions if they do not feel safe, but rather voluntarily engage in tasks based on trust. Second, the findings indicate the need for therapists to be aware of potential alliances ruptures in EFT, and suggest the importance of therapists being sensitive to clients’ needs and preferences at any time in therapy. Some of the ruptures described by the participants indicate that it may be important to identify when clients and therapist have different explanations for the presenting and understandings of the therapeutic tasks (Wampold and Imel, 2015), for example by helping clients with anxiety by presenting specific coping skills to manage symptoms between sessions (Elliott and Shahar, 2018; Watson et al., 2018). Discussing expectations openly in the beginning of treatment may help to tailor the treatment to each individual client, and to monitor potential alliance ruptures during the therapy process. Finally, the participants’ challenges with being transparent about negative experiences in therapy highlight the importance of the therapist being open to negative feedback, particularly negative, and that this open and non-defensive attitude is expressed clearly and unequivocally to the client.

## Data Availability

The datasets generated for this study are available on request to the corresponding author.

## Ethics Statement

This study was carried out in accordance with the recommendations of the Norwegain Regional Committees for Medical and Health Research Ethics with written informed consent from all subjects. All subjects gave written informed consent in accordance with the Declaration of Helsinki. The protocol was approved by the Norwegian Regional Committees for Medical and Health Research Ethics (REK).

## Author Contributions

ØN analyzed the data and wrote the manuscript. P-EB, AH, and SS designed the study, collected and analyzed the data, and wrote the manuscript. ES collected and analyzed the data, and wrote the manuscript. JS designed the study, analyzed the data, and wrote the manuscript.

## Conflict of Interest Statement

The authors declare that the research was conducted in the absence of any commercial or financial relationships that could be construed as a potential conflict of interest.
